# Current and Future Pathways in *Aspergillus* Diagnosis

**DOI:** 10.3390/antibiotics12020385

**Published:** 2023-02-13

**Authors:** Radim Dobiáš, David A. Stevens, Vladimír Havlíček

**Affiliations:** 1 Department of Bacteriology and Mycology, Public Health Institute in Ostrava, Partyzánské Náměstí 2633/7, 702 00 Ostrava, Czech Republic; 2 Institute of Laboratory Medicine, Faculty of Medicine, University of Ostrava, Syllabova 19, 703 00 Ostrava, Czech Republic; 3 California Institute for Medical Research, 2260 Clove Drive, San Jose, CA 95128, USA; 4 Division of Infectious Diseases and Geographic Medicine, Stanford University School of Medicine, Stanford, CA 95128, USA; 5 Institute of Microbiology of the Czech Academy of Sciences, Vídeňská 1083, 142 20 Prague, Czech Republic; 6 Department of Analytical Chemistry, Faculty of Science, Palacký University, 17. Listopadu 12, 771 46 Olomouc, Czech Republic

**Keywords:** aspergillosis, galactomannan, β-d-glucan, PCR, metagenomic next-generation sequencing, lateral flow, bronchoalveolar lavage fluid, serum assays, siderophore, metallophore

## Abstract

*Aspergillus fumigatus* has been designated by the World Health Organization as a critical priority fungal pathogen. Some commercially available diagnostics for many forms of aspergillosis rely on fungal metabolites. These encompass intracellular molecules, cell wall components, and extracellular secretomes. This review summarizes the shortcomings of antibody tests compared to tests of fungal products in body fluids and highlights the application of β-d-glucan, galactomannan, and pentraxin 3 in bronchoalveolar lavage fluids. We also discuss the detection of nucleic acids and next-generation sequencing, along with newer studies on *Aspergillus* metallophores.

## 1. Introduction

*Aspergillus* spp. are saprophytic molds commonly found in the environment. In healthy individuals, several hundred *Aspergillus* spores are inhaled and eliminated daily without harming the host. In susceptible populations, airway colonization by *Aspergillus* spp. may progress to a wide range of respiratory diseases, reflecting the immune status of the host, and exert diverse clinical manifestations. *Aspergillus fumigatus* is the most common species involved, although other species (*A. flavus*, *A. niger*, *A. nidulans*, *A. terreus*, and cryptic species of *A. fumigatus* complex) may be found. Asthma with fungal sensitization (allergic broncho-pulmonary aspergillosis), chronic infections (aspergilloma, chronic pulmonary aspergillosis), and invasive aspergillosis (invasive pulmonary aspergillosis and extrapulmonary disseminated aspergillosis) are the most common presentations.

Among diverse aspergilli, *A. fumigatus* has recently been designated by the World Health Organization as a critical priority fungal pathogen [1]. As mentioned, its spores are spread mainly by the air. For this reason, human airways are the first affected organ at risk of *Aspergillus* infection, especially in individuals with underlying sinus or lung pathology [2]. In particular, persons with impaired natural immunity (e.g., inability to expectorate secretions) or cellular immunity (e.g., neutropenia, impaired T cell immunity, immunosuppressive drugs) are highly susceptible and can suffer from rapidly evolving diseases that may progress to fatal outcomes [3,4]. Therefore, the current most commonly used routine diagnostic methods in immunocompromised or immunocompetent patients must be highly sensitive, specific, and valuable for early diagnosis [5].

## 2. Confirmation of Aspergillosis in a Primarily Sterile Site

The different clinical presentations of aspergillosis have specific demands on diagnostic sampling sites and depend on patient conditions. For example, in invasive aspergillosis (IA) in previously immunocompetent but critically ill patients, or in immunologically compromised patients who may be thrombocytopenic, it is often difficult to obtain a sample from a sterile site to provide sufficient diagnostic information for initiation of antifungal therapy. Current diagnostic protocols used for other aspergillosis disease forms, i.e., chronic pulmonary aspergillosis (CPA) and allergic broncho-pulmonary aspergillosis (ABPA) [5,6], are discussed in the following sections.

### 2.1. Histology, Culture, and Radiography

The speed of IA diagnosis is a critical factor in patient outcomes. Diagnosis is complicated by the fact that *Aspergillus* spp. may be either colonizers or pathogens. The finding of *Aspergillus* spp. from sputum does not necessarily indicate infection, and clinical manifestations of infection may be nonspecific. Our extensive review of the literature reveals the varying predictive value of a sputum culture reported in various populations (Table 1).

Although histology, cytology, or culture evidence from sterile sites may be insensitive (42–81%) or only positive in late stages [7,8], both have still been considered the golden standards in terms of proof of infection [9]. Microscopy performed on tissue specimens and on body fluids does not definitively distinguish *Aspergillus* spp. from other filamentous fungi (particularly *Mucorales*, *Fusarium*, *Penicillium,* and *Scedosporium*), even though essential staining methods such as Gomori’s methenamine silver stain and periodic acid–Schiff, or the application of fluorescent dyes such as Calcofluor white^TM^, Uvitex 2B, or Blancophor^TM^, can provide details on hyphal micromorphology. *Aspergillus* spp. create typically dichotomous and septate hyphae (*Aspergillus* spp. from section *Fumigati*, 45° angle branching hyphae), whereas Mucorales usually show pauci-septate and 90° angle branching hyphae [8]. Sensitivity varies with the specimen source and the laboratory technician’s skills and experience. Finally, delayed culture results or cryptic *A. fumigatus* species identification on special media may compromise early and correct antifungal therapy [10]. Moreover, so-called “cryptic” species (e.g., *Aspergillus lentulus*) closely related to *Aspergillus fumigatus sensu stricto* can hardly be distinguished even after successful culture and require molecular biology tools for differentiation.

Chest radiographs in invasive aspergillosis commonly have nonspecific findings (initially a “ground glass” opacity, proceeding to cavities, nodules, infiltrates, and consolidation), whereas more suggestive findings (“halo” sign, a zone of low attenuation due to hemorrhage and/or edema surrounding a pulmonary nodule; the later “air crescent” sign, a crescent-shaped lucency in the region of the original nodule secondary to necrosis) are present in a third to two-thirds of patients (rarely outside the neutropenic population), are nonspecific (can be seen in other infections, neoplastic diseases, and inflammatory disorders), and can be missed if early and repeated studies are not performed and high resolution computed tomography or other advanced techniques are not employed. The “halo” sign must be distinguished from a nodule margin that is not sharp, the width of the halo should be at least equal to the diameter of the nodule, and at least half of the periphery of the nodule must be surrounded. Both the “halo” and “air crescent” signs are independent predictors of a better outcome, as they appear to correlate with neutrophil recovery. The radiographic presentation can also vary with the underlying disease; an angioinvasive-type picture (discrete nodules) is more common in leukemia patients than in allogeneic stem-cell transplants or others not severely neutropenic, whereas the reverse is true for an airway-invasive picture (more diffuse and scattered findings). Computed tomographic pulmonary angiography may reveal a sign of pulmonary arterial occlusion in invasive mycoses, but there is risk of contrast-induced acute renal injury. Positron emission tomography (PET) scans can be useful in depicting lesions in patients with acute invasive, chronic pulmonary, or allergic diseases.

### 2.2. DNA Analysis and Fungal Species Identification

In some cases, filamentous fungal culture can be complicated and lengthy. Optimized antifungal therapy necessitates correct species identification. Fungal species identification can be verified by the amplification of specific DNA region sequences and identified by sequencing the internal transcribed sequences, calmodulin, β-tubulin, and other partial large subunits of rDNA regions from the young fungal culture. This may lead to targeted antifungal therapy [11].

When fungal elements are detected in formalin-fixed paraffin-embedded (FFPE) tissue sections, and fungus culture is not available, the fungal DNA extracted from FFPE specimens can provide the required answer about the causative agent of the infection. However, the fungal DNA extracted in low concentrations from FFPE samples can be degraded, and the samples often contain substances inhibiting protein digestion or DNA detection and amplification. Fresh non-FFPE tissue samples have shown 97% sensitivity for fungal DNA analysis, while the sensitivity of FFPE specimens was only 68% [12]. Amplification of fungal DNA by a polymerase chain reaction (PCR) combined with DNA sequencing when molds are seen in FFPE tissue has been included in the criteria for Proven Invasive Fungal Disease (molds) Consensus Definitions of Invasive Fungal Disease (IFD) from the European Organization for Research and Treatment of Cancer and the Mycoses Study Group Education and Research Consortium (EORTC/MSGERC) [13].

## 3. Non-Culture-Based Approaches in Bronchoalveolar Lavage Fluid

Obtaining a causative agent, from cultured uncontaminated lower respiratory tract samples, is needed for susceptibility testing [14,15]. However, as previously intimated, a limiting factor is that culturing may take several days to achieve a positive result. Moreover, *Aspergillus* spp. cultures from respiratory tract specimens, in the setting of invasive disease, may have sensitivities as low as 35–63% in sputum or bronchoalveolar lavage fluids (BALFs) [16]. There is a need to distinguish invasive disease from colonization or contamination. The detection of *Aspergillus* antigens or DNA to facilitate appropriate therapy began approximately two decades ago and has been continually optimized.

All BAL studies (including culture) tend to be most useful in the diagnosis of non-leukemic patients and those not severely neutropenic; those patients tend to have an airway-invasive radiographic picture. The diagnostic yield of the assays to be discussed appear to correlate with radiographic lesion size. Any studies (culture, galactomannan, β-d-glucan) that rely on a bronchoscope have to contend with contamination of the bronchoscope in drying cabinets and storage cabinets, resulting in false positivity. In studies comparing sensitivities of BALFs to serum for various tests (not in the same patients), it must be remembered that the populations being studied with BALFs almost assuredly have a higher pre-test probability of being positive (to be submitted to an invasive test) and, conversely, serum testing facilitates serial testing.

### 3.1. Galactomannan

*Aspergillus* cell wall polysaccharide galactomannan (GM) is released in tissues during hyphal development [17] and detected via enzyme-linked immunoassays. For diagnosis of IA, GM detection in BALFs has an excellent predictive value with an optical density index (ODI) between 0.5 and 1.0 [18,19]. In the latest update from EORTC/MSGERC, the upper limit of positivity was shifted to ODI ≥ 1.0 [13]. A positive GM result from BALFs is useful for critically ill non-neutropenic patients suspected of IPA, since tests of GM in their serum are unreliable; negative results are caused by early antigen clearance from the blood due to circulating neutrophils and there is also a problem with substantial false positivity rates [20]. The sensitivity range (81–86%), specificity (88–91%), positive predictive value (PPV, 81%), and negative predictive value (NPV, 94%) were demonstrated in many studies and defined the detection of GM in BALFs as a valuable tool for the diagnosis of invasive pulmonary aspergillosis (IPA) [18,19,21]. Specifically, in non-neutropenic patients, BALF sensitivity outperforms serum GM by about twofold [18]. False-positive GM results in BALF include *Aspergillus* colonization without clinical and radiographic evidence of infection and other non-*Aspergillus* invasive fungal infections [4,22].

CPA patients have relatively high mean ODI values of GM in BALF (ODI = 4.5) in comparison to non-CPA groups (ODI = 0.43) [23]. GM BALF assay sensitivity and specificity for diagnosis across CPA studies has varied between 68 and 77% and 77 and 93%, respectively, and used different cut-off values (ODI = 0.4–1.37) [23,24]. These CPA studies have shown that GM from BALFs is more reliable than GM from serum. Further, when the cut-off was high (i.e., ODI = 2.5), higher test specificity (100%) for the diagnosis of CPA was achieved [24]. Some aspects of GM application in BALFs have remained unclear. For example, a sputum study reported many false-positive GM results [25].

### 3.2. 1,3-β-d-Glucan (BDG)

The polysaccharide fungal cell wall component, BDG, is a panfungal biomarker. BDG may be involved in the mechanism of pulmonary inflammation in patients with acute fungal pulmonary hypersensitivities [26]. BALF from ABPA patients showed high BDG concentration, which may enhance the expression and release of cytokines through nuclear factor (kB) activation in respiratory epithelial cells [27]. BDG elevations in BALF will occur in a wide spectrum of pulmonary diseases and will not be useful in the differential diagnosis of IPA as a single diagnostic test. Using a positive cut-off ≥ 80 pg/mL, 54–57% and 38–83% IPA sensitivity and specificity in non-neutropenic and neutropenic patient populations could be achieved [28,29]. In contrast, the combination of the BDG and GM assay on BALFs has the highest diagnostic odds ratio, as shown in a study on patients with CPA. The CPA diagnostic sensitivity and specificity of BALF GM and BDG were 78% and 90% and 78% and 73%, respectively [30].

### 3.3. Aspergillus-Specific PCR, Aspergillus DNA in Panfungal PCR Assays, and Metagenomic Next-Generation Sequencing

Numerous molecular methods are available to detect and identify *Aspergillus* spp. in BALFs. DNA- and RNA-based techniques have potential but are not implemented in most diagnostic laboratories [9]. In IPA diagnosis, in-house and commercially available sets, including multiplex real-time PCR analysis, showed a sensitivity and specificity range of 40–89% and 69–99%, respectively, across a broad spectrum of patients [15,31,32,33,34,35]. A problem in assessing PCR results in reports is the great variability in the techniques employed.

During IPA diagnosis, combining at least two different methods, especially GM and PCR for *Aspergillus* in BALFs, can increase sensitivity and specificity up to 83 and 95%, respectively [3,16,33]. However, the concordance between positive GM and PCR in BALF in patients with and without IPA is significantly lower (*p* < 0.001) in ICU patients (32%; 43% in COVID-19 patients, 18% in non-COVID-19 patients) than in the classically immunocompromised (92%) [35]. Promising data from *Aspergillus* PCR in BALF studies in the diagnosis of aspergillosis are somewhat limited [3,9,16,17]. On the other hand, *Aspergillus* PCR from BALF was included as one of the mycological evidence criteria of probable IPA in the last update from EORTC/MSGERC [13].

Recently, metagenomic next-generation sequencing (mNGS) has been used as a modern approach providing molecular-based fungal DNA evidence in diagnosing suspected pneumonia. Ideally, sequencing could reveal genus and species and provide information about possible drug resistance. However, the BALF application of mNGS on groups of immunocompromised patients identified more viral pneumonia but had much lower diagnostic accuracy for fungal infections (99% vs. 77%). Reduced sensitivity to fungal infection was primarily due to low sensitivity to IPA [36].

### 3.4. Pentraxin 3 Is a Host Factor

**Figure 1:**
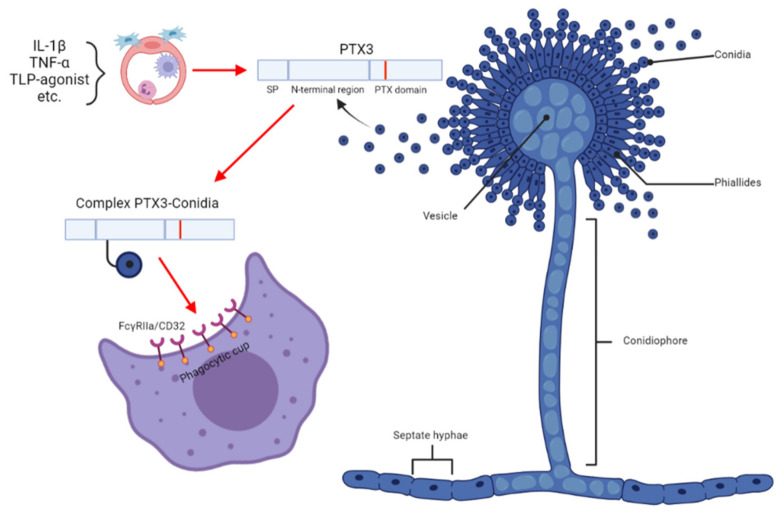
The role of Pentraxin 3 in the *Aspergillus* conidial model. IL-1β, interleukin 1-β; TNF-α, tumor necrosis factor α; FcγRIIa/CD32 proteins (also known as CD32 in the Cluster of Differentiation) are activating-type Fc receptors, the FcγRII family are active as receptors for immunoglobulins, and FcγRIIA acts as a receptor for pentraxins. The image was generated with Biorender software (https://biorender.com/ (accessed on 1 January 2023)).

Contrary to all tools described in Section 3.1, Section 3.2 and Section 3.3, the monitoring of pentraxin 3 (Ptx3) in the BALF of patients with IPA represents the application of a human host factor in the diagnosis of IPA [37]. Ptx3 is a mammalian plasma-soluble receptor that is a non-specific human proinflammatory biomarker and is synthesized in the endothelium and macrophages, neutrophils, fibroblasts, and other immune cells (Figure 1) [38]. Although Ptx3 serum level is considered a nonspecific feature with respect to invading pathogens, Ptx3 monitoring in BALF, i.e., in the early and primary site of infection, produces interesting results. In a recent study, the application of Ptx3 in BALFs enabled IPA to be distinguished from CPA, bacterial invasive infection, or fungal colonization [37]. Median Ptx3 concentrations in patients with and without aspergillosis were 4545.5 and 242.0 pg/mL, respectively (95% CI, *p* < 0.05). The optimum Ptx3 cutoff for IPA was 2545 pg/mL, providing a sensitivity, specificity, positive predictive value (PPV), and negative predictive value (NPV) of 100, 98, 95, and 100%, respectively. Of note, a Ptx3 assay in combination with siderophores (see Section 8) was used to successfully distinguish IPA from invasive mucormycosis [37].

## 4. Monitoring of Serum Biomarkers

The possibility of serum testing for early *Aspergillus* spp. biomarkers is highly desirable since blood sampling is easy to perform and can be more reproducible than BALF sampling with computed tomography assistance. These non-culture-based assays are often combined with microscopy or culture [15].

### 4.1. Galactomannan

GM is the major and historically most popular serum biomarker. Its mycological importance in IPA diagnostics has been recognized by EORTC/MSGERC criteria [13,15]. In serum samples of patients with hematological malignancies not receiving antifungal prophylaxis, use of a GM ODI cut-off of 0.5 resulted in high sensitivity (78–79%) and specificity (85–86%) [15,17]. Early and repeated testing is key to obtaining useful results. When this is performed in monitoring during immunosuppression or at the appearance of nonspecific symptoms, a positive result can precede a diagnostic finding from plain chest radiography, computed tomography of the lungs, or a positive culture 80% of the time. Antifungal therapy, or walled-off infections, decreases the sensitivity of the test; dietary galactomannan (particularly liquid nutritional supplements), graft-vs.-host autoreactive antibodies, some antibacterials (particularly those produced by *Penicillium*), myeloma, and multiple transfusions can cause false positivity, and false positives are much more common in children. The course of repeated assays after a diagnosis is confirmed can be useful as clearance of positivity correlates with recovery of immunity and with a favorable course; failure to clear, or a rising positive titer, has a bad prognosis.

Positivity is much lower in solid organ transplants with pulmonary aspergillosis. In the non-neutropenic population, IA incidence ranges from 0.3 to 5.8% in the critical care units and is characterized by low serum GM predictive value [20] and sensitivity attenuated to 42% [18,20]. In CPA, serum GM decreased to very low sensitivity, specificity, PPV, and NPV, which were 23, 85, 60, and 50%, respectively. Thus, the serum GM assay cannot be used for CPA diagnosis [39,40].

### 4.2. 1,3-β-d-Glucan

BDG may be released into the blood during invasive infections triggered by *Aspergillus*, *Candida*, *Saccharomyces*, *Fusarium*, *Trichosporon*, *Acremonium*, or *Pneumocystis jiroveci*. This also occurs in phaeohyphomycosis, chromoblastomycosis, and other invasive mold infections [41,42,43,44], although there is very limited utility in cryptococcosis and infection by *Mucorales*. Assay sensitivity and specificity depend on which fungal pathogen is involved and the underlying patient populations examined [9,43].

If serum GM and BDG are used alone in the diagnosis of IA, they have lessened utility. However, improvements in sensitivity and NPV are achieved by “at least one positive” analysis with the GM and BDG assays, with the sensitivity, specificity, PPV, and NPV being 85, 70, 71, and 84%, respectively [41]. When a cut-off of GM ODI = 0.5 is used, the combined sensitivity and specificity for serum GM is 74% and 85%, respectively.

Serum *Aspergillus* PCR in the diagnosis of IA has been reported to have a mean sensitivity of 80.5% and a mean specificity of 78.5%, and BALF *Aspergillus* PCR has been reported to have a mean sensitivity of 90.2% and a mean specificity of 96.4%; serum BDG alone has a sensitivity of 81% and specificity of 61% [5]. With any assay, the pre-test probability characteristics for the population under study will affect these indices. The probability of a suspected IA diagnosis will increase with several positive tests in a patient.

The serum sensitivity of BDG in CPA diagnosis is very low (~20%) [39]. However, the possibility of combining GM and BDG assays in BALF was reported. The study had higher diagnostic accuracy with a cut-off of ODI = 0.5 (GM) and 100 pg/mL (BDG), with a sensitivity and specificity of 78 and 90%, respectively.

### 4.3. Aspergillus-Specific PCR and Metagenomic Next-Generation Sequencing

Efficient DNA extraction, validated protocols of amplification, and overall good performance are crucial for the successful analysis of *Aspergillus* DNA from blood derivatives [17]. PCR screening of serum for *Aspergillus* in patients at high risk of invasive fungal disease should be considered; a negative result has a high negative predictive value, enabling the invasive fungal infection to be excluded [16]. Based on systematic reviews, *Aspergillus* PCR methods with blood were included as one of the mycological evidence criteria of probable IPA in the last update of Consensus Definitions of Invasive Fungal Diseases from EORTC/MSGERC [13]. Many recent studies and meta-analyses have revealed the detection of DNA by *Aspergillus* PCR, with a sensitivity of ~88% and a specificity of ~75% for a single PCR positive result; two consecutive positive PCR tests showed a sensitivity of ~75% and a specificity of ~87% [45,46,47,48,49,50,51]. In studies, the serum PCR assay has preceded diagnosis on the basis of GM or BDG by 4–6 days.

Detecting the cell-free DNA of *Aspergillus* spp. via peripheral blood mNGS is a rapid and non-invasive method for capturing a wider spectrum of *Aspergillus* pathogens in one reaction and can be used to predict IPA [52]. However, based on a multicenter retrospective cohort study [53], there was no clinical impact in 71 cases with 82 mNGS tests. The clinical impact of mNGS to diagnoses of IPA is presently limited and further studies to define the role of mNGS in current testing algorithms are needed. On the other hand, commercialization of the plasma cell-free DNA approach and its niche uses will undoubtedly accelerate the growing field of infectious disease diagnostics [53].

## 5. The *Aspergillus*-Specific Lateral Flow Device

An extracellular glycoprotein secreted by *Aspergillus* spp. during active growth can be detected by a point-of-care test, the *Aspergillus*-specific lateral flow device (LFD). This relies on immunochromatography, which is a capillary action that results in the capture of antigens by a murine monoclonal antibody fixed to a nitrocellulose membrane. The LFD may be applied to serum and BALF. BALF testing in probable/proven IPA in an overall population of mixed patients (solid organ transplant, ICU, respiratory disease, hematologic malignancies) had sensitivity, specificity, PPV, and NPV of 73, 90, 61, and 94%, respectively [54]. A pooled sensitivity of 68% and specificity of 87% for differentiating proven or probable IA compared to a non-IA control group in serum samples was noted across several studies [55,56,57]. Studies have suggested positive tests in serum may precede GM or BDG positivity. Overall, LFD testing still needs further multicenter studies to receive a recommendation for clinical routine practice.

## 6. Antibodies

Despite the elevated levels of IgA and IgG-specific *A. fumigatus* in infected patients, antibody detection tests are not recommended for the diagnosis of IA [41]. Instead, serum samples may be useful in patients with pulmonary cavities or nodules of unknown origin predefined by imaging methods [17,58]. Antibody detection kits have excellent performance in diagnosing CPA and ABPA. Patients with suspected ABPA can be diagnosed by elevations of total immunoglobulin E (IgE) and *Aspergillus*-specific IgE in combination with culture from the lower airways or clinical symptoms [9,17,39,59]. IgE against recombinant (rAsp) *Aspergillus* antigen f1 (sensitivity, 89%; specificity, 100%) or the combination of either rAsp f1 or f2 *Aspergillus* antigens (sensitivity, 100%; specificity, 81%) are the best tests for differentiating ABPA from *Aspergillus*-sensitized asthma [60].

## 7. Gliotoxin and Bis(methylthio)gliotoxin

Gliotoxin (Gtx) and bis(methylthio)gliotoxin (bmGtx) are extracellular toxins secreted by *A. fumigatus* under stress conditions [61] and have been proposed as potential IPA biomarkers [62]. The diagnostic value of both toxins in serum or BALF remains inconclusive, as some reports do not recommend Gtx/bmGtx to be used in diagnosing IPA [63]. Even during in vitro production, Gtx and bmGtx are differentially diffused into the extracellular media during *A. fumigatus* infection regardless of the growth format tested [64].

In general, proper site selection for bodily fluid collection (proximal or distant fluids) is a prerequisite for successful detection. In a recent study with urine samples [65], a Gtx/creatinine index recorded in 13 patients with probable aspergillosis provided 46.2% detection sensitivity (95%CI 19.2–74.9%) and 100% specificity (95%CI 84.6–100%). No bmGtx was seen in the patients’ specimens.

Gtx is produced by various aspergilli, e.g., by *A. lentulus*, *A. udagawae*, and *A. viridinutans*. In *A. fumigatus,* the release of Gtx is the most prominent. Compared to siderophores (Section 8), Gtx specificity in *A. fumigatus* diagnosis is lower, as related molecules are produced by various *Candida* spp. and by species of the fungal genera *Penicillium, Scedosporium,* and *Alternaria*. Stress factors inducing Gtx release by *A. fumigatus* may also be based on nutrients. As an example, zinc deficiency upregulated the expression of GliZ, which encodes a Zn2-Cys6 binuclear transcription factor that regulates the expression of genes required for Gtx synthesis [61]. Gliotoxin production was decreased inversely proportional to zinc concentration, and the same result was investigated in the absence of ZafA, a zinc-dependent transcription activator.

## 8. *Aspergillus* Metallophores

Microorganisms have developed the ability to produce metallophores, which are chelators capable of scavenging metals from hostile environments and transporting essential nutrients into microorganisms to promote their growth, especially in settings where metals are limited [66]. Metallophores (called siderophores if acquiring iron) have diagnostic potential in mammalian hosts. In the complex array of defense mechanisms, the host fights back through the secretion of mammalian siderophores (which includes dihydroxybenzoic acids) and proteinaceous lipocalins [67]. In the battle for nutrients, more successful pathogens produce stealth siderophores, thus escaping recognition by lipocalins due to varied siderophore structures and maintaining their chelating function [68].

**Figure 2:**
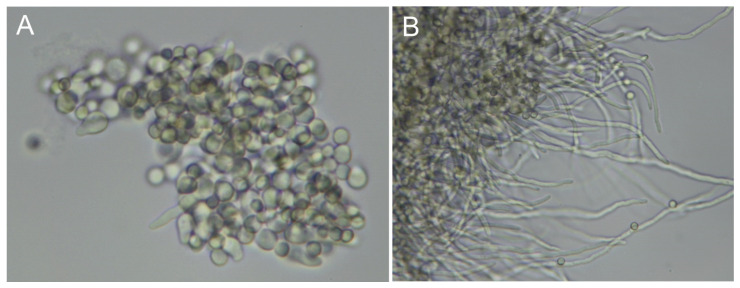
Asynchronous germination of *A. fumigatus* conidia. Fungal hyphae development 7 h (**A**) and 10 h (**B**) postinoculation. The time course of conidial germination in flask cultures was recorded by bright-field microscopy at 60× magnification.

In *Aspergillus*, siderophores are produced in different phases of fungal development, from conidial germination to the stage of mycelial growth. Germination is induced by micronutrient stimuli (carbon, nitrogen, metals) and can be asynchronous [69]. The variable number of conidia and developed hyphae at different time windows of *A. fumigatus* germination should be noted (Figure 2). As a result, the effective appearance rates (number of molecules per fungal cell per time interval) may vary in different fungal phenotypes, and one can experience increases (during mycelial growth) or declines (autolytic phase) in the corresponding growth curves [65].

Fungal siderophore applications in positron emission tomography and computed tomography (PET/CT) imaging have recently been reviewed [70]. Notably, the response to antifungal therapy when monitoring the progression and siderophore decline of *A. fumigatus* infection was reported in neutropenic rats [71]. In that work, ^68^Ga-TafC and ^68^Ga-ferrioxamine E (FOXE) were used to monitor infection progression and treatment, respectively. *A. fumigatus*-infected rats treated with posaconazole showed a rapid decrease in ^68^Ga-FOXE uptake in the lungs on subsequent PET/CT scans after the first positive ^68^Ga-FOXE lung scan [71].

Of note, PET/CT represented the technology that originally predicted siderophore secretion into host urine [72]. On this seminal finding, the later concept called infection metallomics was built [73]. The concept is based on Fourier-transformed ion cyclotron resonance mass spectrometry and isotopically resolved data filtering [74] and has been applied in diagnostic work based on various siderophores [37,65,75,76,77,78,79].

*Aspergillus* siderophores were found in the urine, sputum, BALF, and breath condensate of infected patients [70], whereas they were absent or at low levels in patients that were only colonized [37,65,70,76]. Under favorable conditions, the top segments of metabolite secretion curves are reflected in patients’ bodily fluids. Merely one can detect only the “tip of the iceberg”, as the fungal extracellular secretome is diluted in body circulation and the onset or decline of the infection is buried in chemical background noise [73].

Of note, none of the two intracellular and two extracellular *A. fumigatus* siderophores [80] are secreted by a mammalian host; thus, the specificity of siderophore-based diagnostics may reach 100% [70]. Sensitivity can also be very high due to favorable metabolite-specific synthesis [73,81] and renal excretion rates [72,82]. Non-invasive sampling thereby represents an attractive aspect of siderophore application, and there is less chemical “background noise” in the urine compared to host serum, as shown in an animal model [78] and in humans [65]. All these analytical factors make siderophores prospective fungal biomarkers in the window of diagnostic opportunity [76], which results in prompt and correct antifungal therapy. Factoring urine siderophore levels as a function of creatinine secretion eliminates aberrant results caused by declines in renal function in acutely ill patients [83].

In a recent clinical study of 13 patients diagnosed with probable IPA, the transition from colonization to the *A. fumigatus* invasive stage was shown to be accompanied by secretion of TafC, triacetylfusarinine B (TafB), and ferricrocin (Fc) siderophores into urine, with strikingly better sensitivity performance compared to serum sampling [65]. The TafC/creatinine index, with a median value of 17.2, provided 92.3% detection sensitivity (95%CI 64.0–99.8%), 100 % specificity (95%CI 84.6–100%), and was the best-performing index (substantially better than the corresponding results provided by GM and BDG serology in the same patient cohort). TafC copy numbers in human urine were higher than those of Fc. Though invasive aspergillosis can be distinguished from colonization based on siderophore profiles [65], the current data indicate that distinguishing IPA from CPA assaying siderophores may not be possible [37].

In the One Health application of siderophores, the diagnosis of aspergillosis was recently elaborated in horses. In two equine cases of guttural pouch aspergillosis, panfungal Fc, i.e., an intracellular siderophore, was detected in debridement [75]. *A. nidulans* and *A. fumigatus* were independently confirmed by culture, fungal spp. DNA, GM, and microscopy. Of note, Gtx was detected in the IPA equine case, specifically in the BALF and lung tissue samples (86 ng/mL and 2.17 ng/mg, AUC = 1).

The importance of studying the complete microbial growth curve was emphasized with the application of siderophores in a mycovirus–fungal host functional study [81]. It has been shown that improper selection of isolated time points (instead of assaying the complete curve) may lead to conflicting interpretation results. Since the secretion of fungal virulence factors is growth phase-dependent, this aspect must also be considered in fungal diagnostics. Repeated assays of patient samples may be needed.

In *A. fumigatus,* dual-purpose isocyanides represent another group of metallophores [84]. Demonstrating the potential application of these copper-containing fungal metabolites (termed chalkophores) in microbial diagnostics awaits further studies.

## 9. Conclusions

We conclude with the authors’ opinions. Recent studies that may have promise but which were not discussed above include the detection of *Aspergillus* antigens (GM-like) in urine and breath analysis of volatile metabolites. Compared to next-generation sequencing, PCR with specific DNA probes, galactomannan, and other serology tools, fungal secondary metabolites can provide better indications of the status of live fungal cells with active secondary metabolism rather than circulating pieces of dead fungal bodies or other products. False positivity is excluded as mammalian cells do not synthesize these molecules. False negative rates are lower than those obtained with DNA sequencing and serology since small molecular weight biomarkers have much higher tissue–blood barrier transmission and renal clearance rates than high molecular weight nucleic acid or polysaccharide polymers. GM and BDG are not chemical entities with a single molecular weight. As a result, their transfer through body barriers is associated with analytical losses, and they may be bound to other molecules that obscure their detection. Microbial secondary metabolites, on the other hand, are small molecules that may sometimes appear in urine in high copy numbers, facilitating detection.

## Figures and Tables

**Table 1:** Predictive value of positive *Aspergillus* sputum cultures for invasive aspergillosis.

Risk Group	*Aspergillus* Infection, %
Acute leukemia	100
Solid cancer	0
Neutropenia only	94
Neutropenia or bone marrow transplant	72
Bone marrow transplant only	82
Corticosteroids	63–65
Antibiotics	39
HIV	14
Solid organ transplant	56
Chronic obstructive lung disease	15–22
Non-neutropenic, mechanically ventilated, ICU	20

## Data Availability

Not applicable.
